# Design requirements of upper extremity supports for daily use in Duchenne muscular dystrophy with severe muscle weakness

**DOI:** 10.1177/20556683241228478

**Published:** 2024-02-08

**Authors:** Suzanne J Filius, Jaap Harlaar, Lonneke Alberts, Saskia Houwen-van Opstal, Herman van der Kooij, Mariska MHP Janssen

**Affiliations:** 1 Department of Biomechanical Engineering, 2860 Delft University of Technology, Delft, the Netherlands; 2 Department of Orthopaedics and Sports Medicine, Erasmus Medical Center, Rotterdam, the Netherlands; 3 Department of Rehabilitation, Donders Institute for Brain, Cognition and Behaviour, 6034 Radboud University Medical Center, Nijmegen, the Netherlands; 4 Amalia Children’s Hospital, Nijmegen, the Netherlands; 5 Department of Biomechanical Engineering, University of Twente, Enschede, the Netherlands; 6 Rehabilitation Center, Klimmendaal, Arnhem, the Netherlands

**Keywords:** Upper extremity, arm support, exoskeleton, orthosis, Duchenne muscular dystrophy

## Abstract

**Background:**

People with Duchenne muscular dystrophy (DMD) cope with progressive muscular weakness and consequential upper extremity function loss. They benefit from arm supports, or arm exoskeletons, to assist arm function. Especially for severe muscle weakness (DMD ≥ Brooke Scale 4), the design of such arm support is challenging. This study aims to structurally develop functional and technical design requirements of arm supports for people with DMD Brooke Scale 4.

**Methods:**

An overview of clinical characteristics and a classification of clinically meaningful activities were derived from data from the Dutch Dystrophinopathy Database and available literature. Based on these, functional and technical design requirements of arm supports were developed and matched to the achievable needs of the user.

**Results:**

First, the clinical characteristics of the target population, such as strength, range of motion, and functional ability, are given. Next, clinically relevant activities of daily living are translated to functional requirements categorised in a ‘must,’ ‘should,’ and ‘could’ category. Last, the technical requirements to realise these functional goals are presented.

**Conclusions:**

The recommendations following from the functional user needs, technical requirements, and safety considerations can be used to make the development of assistive arm supports for people with DMD Brooke Scale 4 more user-centred.

## Introduction

### Muscular dystrophy affecting upper limb

Duchenne muscular dystrophy (DMD) is a progressive neuromuscular disease (NMD) caused by a dystrophin gene mutation that results in a lack of the dystrophin protein. An absence of dystrophin makes the muscle cells highly vulnerable to stress during muscle contraction.^1^ As a result, the muscles of DMD patients weaken over time. DMD is often diagnosed around the age of 5,^2^ and around the age of 10, DMD patients start using a wheelchair and cope with loss of upper extremity function.^3^ At present, no cure has been found, but since 1960, life expectancy has increased from around 14 years to over 39 years due to medical interventions such as corticosteroid use and (eventually) mechanical ventilation.^4^ Especially for wheelchair users, loss of upper extremity function has a great impact on their independence, social participation and quality of life.^5–7^ Since the timespan that DMD patients make use of a wheelchair becomes longer, it becomes more important to focus on possibilities to support functions of the upper extremity.

### Intended target population

**Figure 1.**
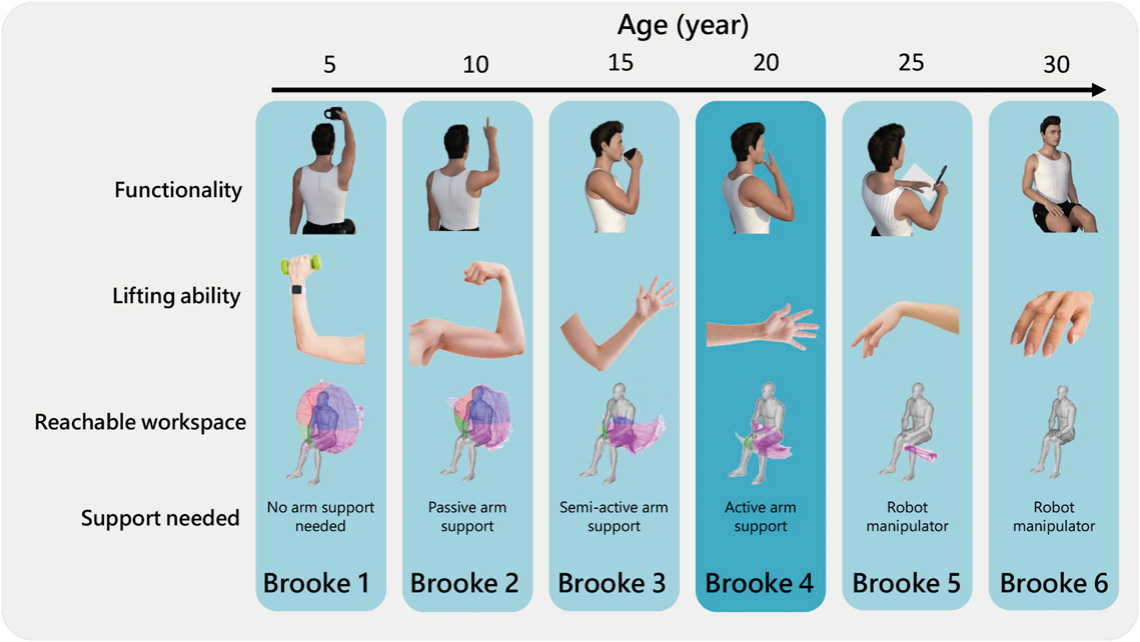
Summary of the functional user characteristics classes per Brooke scale. Range of motion images are retrieved and adapted from Han et al.^9,76^ Functionality models were retrieved from DAZ Productions.^52^ Lifting ability figures were retrieved from Internet [vecteezy.com; pexels.com; splash.com].

Figure 1 summarises the general characteristics of DMD patients per Brooke Scale^8^ to highlight how the selected population fits within the spectrum of DMD. In this paper, we will specifically focus on patients within Brooke Scale 4 (ie, “can raise hands to the mouth, but cannot raise an 8 oz [∼230 g] glass of water to the mouth”^8^). This population is often in the late non-ambulatory stage.^2^ About 4%–10% of the DMD patients have an upper extremity classification of Brooke Scale 4,^9–15^ over 25,000 patients worldwide. We focused on this population since these patients are often too weak to use a non-motorised arm support.^6^ However, external robotic manipulators are not intuitive and potentially worsen disease progression by taking over the execution of tasks completely, contributing to disuse. These specific functional needs contribute to the lack of arm support availability for this population. This will be further discussed in the next section.

### Availability of arm supports

Compensation for the weight of the arms can reduce the net joint moments to perform activities of daily living (ADL) to benefit people with arm disabilities.^16^ Over the past century, many attempts have been made to design supportive devices for people with arm disabilities.^5,17^ Currently, passive (ie, non-motorised weight support through springs or counterweights) and semi-active (ie, passive weight support with motorised adjustment) systems are commercially available.

A major functional limitation of passive systems is that they do not support the weight of the arms over the entire workspace equally. The level of support determined by device provision is often set to function well in the frontal and horizontal workspace. As the disease progresses, it becomes too difficult to raise the arms above the head, lift objects,^18^ and perform downward movements with passive arm supports.^6,19^ Semi-active systems allow the users to adapt the support level for the required workspace by using a button with the contralateral arm,^6^ making the interaction cumbersome.

Besides passive and semi-passive arm support systems, external robotic manipulators are commercially available. These are active systems controlled by a joystick for endpoint control of the manipulator that overtakes the function of the human arm. These devices interact directly with the objects in the environment without the human arm. Robotic manipulators might be a solution when no or very limited passive range of motion (pROM) is left (eg, due to shortened muscle and joint contractures), often seen in higher disease stages (Brooke Scale ≥5). However, it has been shown that physical arm training slows down the progression and prevents contractures that may develop from disuse.^20^ So, as long as the pROM is sufficient, it is essential to keep the arm muscles involved and provide assistance as needed.

Unfortunately, the authors are unaware of commercially available systems appropriate for the severe disease stages, ie, Brooke Scale 4 in DMD, encompassing and involving the human arm in the motion. Therefore, there remains a need for dedicated assistive arm support to be developed, with specific functional and technical design requirements. These requirements should be based on the clinical characteristics of people with DMD classified in Brooke Scale 4, focusing on the most meaningful and feasible upper extremity tasks.

### Aim

This paper aims to develop functional and technical arm support design requirements for people with DMD classified in Brooke Scale 4.

## Methods

The data presented in this study are based on PubMed prior to October 2022, reference snowballing and data from the Dutch Dystrophinopathy Database (DDD).

### Data collection

The DDD is a national register for Duchenne and Becker muscular dystrophy patients in the Netherlands. The database contains natural history data collected from annual clinical care assessments. In the database, 39 DMD patients with Brooke Scale 4 are included. Access to this database was granted by the Duchenne Centre Netherlands (DCN), a collaboration between the Leiden University Medical Center (LUMC), the Radboud University Medical Center (Radboudumc), Kempenhaeghe-MUMC+, the Duchenne Parent Project (DPP) patient organisation and ‘Spierziekte Nederland’ (SN) patient association. We requested data for muscle strength, range of motion (ROM), and performance of upper limb (PUL) scores.

### Literature search

For the characteristics of the intended target population and the functional requirements, literature was mainly searched using a combination of the terms (or their synonyms) DMD, upper extremity, muscle force/torque, active range of motion (aROM) and pROM, joint impedance, reachable workspace, and PUL. Only papers in which patients with Brooke Scale 4 participated or where it could be determined what data corresponded with patients in Brooke Scale 4 were included. More literature has been published on these characteristics in DMD. Yet, in several papers, the clinical characteristics of the patients are not categorised per Brooke scale but based on age or other functional scales. For the literature search of the technical requirements, additional search terms were added: ADL, angular velocities, arm support and its accompanying synonyms (eg, exoskeleton, assistive device, dynamic arm supports, orthosis). Also motorised upper extremity arm supports for ADL used in different pathologies (eg, stroke, incomplete spinal cord injury) were included. Not all data we found in the literature could directly be used for our results. Therefore we used additional literature and made several assumptions to interpret the data. For example, literature-based assumptions on anthropometrics and body weight were used for recalculating forces to joint torques or to estimate arm segment weights.

### Functional requirements and arm model definitions

To state the functional requirements, the ADLs that are identified as clinically meaningful for DMD (ie, high-level functional requirements) were first categorised into a ‘must,’ ‘should,’ and ‘could’ category based on an estimated required strength and workspace score. Next, we dissected these activities into ‘low-level functional requirements’ (ie, ROM, velocity and support level) by brief analyses of the required ROM and support level. For the support level analysis, a custom kinematic rigid body model of the arm was created to estimate the internal shoulder and elbow joint moments for a set of ADL poses. The segment parameters used for this kinematic model were taken from Veeger et al.^21^

#### Arm model

**Figure 2.**
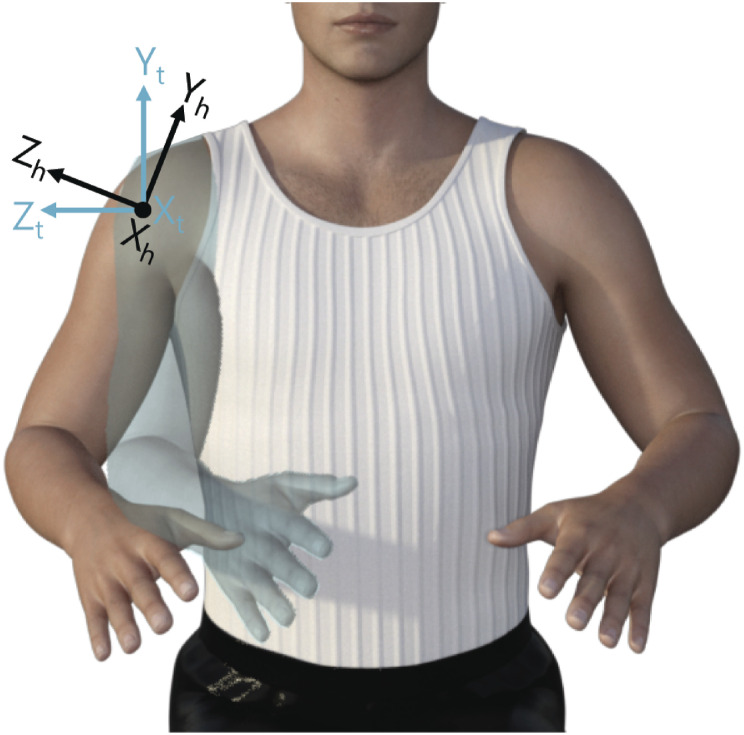
Representation of the thorax (t) and humerus (h) coordinate systems according to the ISB recommendations.^22^ The x-axis points outwards of the paper. The human model shows elevation rotation in the frontal plane around *x*_*h*_ with respect to the thorax coordinate system.^24^

The custom kinematic rigid body arm model followed the ISB recommendations^22,23^ with adaptations of Stienen and Keemink^24^ to define the joint rotations of the arm. Within these definitions the wrist joint is approximated by three axes of rotation (sequence flexion/extension, ulnar/radial deviation, pronation/supination), the elbow joint is approximated by a hinge joint (flexion/extension) and the shoulder joint as ball-and-socket joint relative to the thorax^23^ with three axes of rotation (sequence horizontal rotation, elevation rotation, axial rotation). Where *horizontal* rotation, also referred to as ‘(angle of) plane of elevation,’ is the rotation around the y-axis fixed to the thorax coordinate system, *elevation* is the rotation around the x-axis fixed to the humerus coordinate system. *Axial *rotation is the rotation around the y-axis fixed to the humerus coordinate system, see Figure 2.

In literature, the classical medical definition (ie, shoulder ab-/adduction, flexion/extension, internal-external rotation) is often used. For the ROM analysis, we translated, when possible, the medical definition into the ISB recommendations.

## Results

### Characteristics of the intended target population

This part of the paper overviews the patient characteristics commonly described in DMD patients with Brooke Scale 4. Table 1 describes data on upper extremity muscle strength, ROM and the functional ability of this population. The average age of the DMD patients with Brooke Scale 4 within this overview is approximately 15 years (5–29 years).

**Table 1.** Characteristics of DMD patients in Brooke Scale 4.

Author	Year of publication	Nr. DMD Brooke 4	Outcome as Average (reported measure on data variability)
Muscle strength^a^			
Brussock et al.^25^	1992	1	^b^Shoulder abduction: R = 2.9 N, L = 3.4 N Elbow flexion: R = 2.9 N, L = 2.9 N Elbow extension: R = 2.9 N, L = 2.9 N
Janssen et al.^3^	2017	3	Shoulder abduction: R = 15 N (95% CI 3;26 N); 5.6 Nm (95% CI 4.9;6.2 Nm) Elbow flexion: R = 7 N (95% CI -3;17); 1.7 Nm (95% CI −1.2;4.5 Nm) Elbow extension: R = 8 N (95% CI 2;14); 1.8 Nm (95% CI −.4;4.0 Nm)
Dutch Duchenne Database	2022	4	Shoulder abduction: R = 5.1 N (range 0–10.5 N) Elbow flexion: R = 18.1 N (range 9.3;35.3 N) Elbow extension: R = 25.4 N (range 17.3–41.2 N)
pROM			
Dutch Duchenne Database	2022	36	^b^Shoulder flexion: R = 150° (range 70;180); L = 146° (range 70;180). Shoulder abduction: R = 145° (range 70;180); L = 145° (range 70;180) Elbow flexion: R = 141° (range 120;150); L = 142° (range 110;155) Elbow extension^c^: R = 23° (range −20;85); L = 27° (range −20;90)
Janssen et al.^3^	2017	3	Shoulder abduction: R = 128° (95% CI 97°;160°) Elbow flexion: R = 135° (95% CI 114°;155°) Elbow extension^c^: R = 50° (95% CI −45°;146°)
aROM			
Janssen et al.^3^	2017	3	Shoulder abduction: R = 0° (95% CI 0°;0°) Elbow flexion (against gravity): R = 110° (95% CI 16°;204°) Elbow extension^c^ (with gravity): R = 40° (95% CI −24°;104°)
Reachable workspace (RSA^d^)			
Han et al.	2015	4	RSA right and left = .090 (SD 0.061)
Corrigan and Foulds^26^	2020	5	RSA right = .0858 (range .025;0.165) RSA left = .0226 (range .011;0.037)
Janssen et al.^5^	2021	1	RSA right = 0.2 RSA left = .17
PUL (1.2)^e^ [% of max]			
Gandolla et al.^27^	2020	4	PUL total = 56% (range 46;64%) PUL shoulder dimension = 0% (range 0;0%) PUL elbow dimension = 59% (range 38;71%) PUL wrist and finger dimension = 91% (range 88;96%)
Janssen et al.^3^	2017	3	PUL total = 43% (95% CI 28;58%) PUL shoulder dimension = 0% (95% CI 0;0%) PUL elbow dimension = 29% (95% CI 12;44%) PUL wrist and finger dimension = 83% (95% CI 63;108%)
PUL (2.0)^e^ [% of max]			
Pane et al.^28^	2018	28^f^	PUL total = 36% PUL shoulder dimension = 0% PUL elbow dimension = 29% PUL wrist and finger dimension = 77%
Janssen et al.^5^	2021	1	PUL shoulder dimension = 6% PUL elbow dimension = 65%
Cruz et al.^29^	2020	4^g^	PUL total = 36% (range 29;45%) PUL shoulder dimension = 0% (range 0;0%) PUL elbow dimension = 29% (range 12;41%) PUL wrist and finger dimension = 77% (range 69;92%)
Dutch Duchenne Database	2022	21	PUL total = 38% (range 29;50%) PUL shoulder dimension = 0% (range 0;0%) PUL elbow dimension = 35% (range 18;53%) PUL wrist and finger dimension = 77% (range 69;92%)

^a^Muscle strength values consist of both force and torque, depending on the data collection or reporting method. If handheld dynamometry is used, force (N) is often reported while torque is measured. Moment arms, however, are usually not reported.

^b^Where shoulder abduction in the classical medical definition is defined as elevation rotation with 0° horizontal rotation, and shoulder flexion is defined as elevation rotation with 90° horizontal rotation.

^c^Note that a positive value for elbow extension means that there is no full elbow extension, see Figure 4.

^d^The Relative Surface Area (RSA) ranges between .0 and 1.0, where 1.0 corresponds to the reachable workspace envelope of the entire frontal hemisphere the subject reached.

^e^PUL refers to the Performance of Upper Limb scale, a functional scale to measure upper extremity function.^30^ There are two versions of this scale. The PUL 1.2 has a maximum score of 74 (16 for shoulder dimension, 34 for elbow dimension and 24 for wrist and finger dimension), The PUL 2.0 has a maximum score of 42 (12 for shoulder dimension, 17 for elbow dimension and 13 for wrist and finger dimension).

^f^Number of participants is based on both Brooke Scale 3 and 4, so number of participants with solely Brooke Scale 4 is not available. The data presented, however, is only from patients with Brooke Scale 4. No data on variability between subjects is available.

^g^Not clear if Brooke Scale 4 and 5 were included or Brooke Scale 4 alone.

#### Muscle strength

When designing an arm support, it is essential to know how much joint torque patients can provide to a varying degree. We found three sources that described the muscle strength of in total eight patients in Brooke Scale 4 (see Table 1). Different methods were used to measure muscle strength. Unfortunately, in most sources, only force and no joint torques were reported. Brussock et al.^25^ and Janssen et al.^3^ used a fixed-frame dynamometer to measure strength, while a hand-held dynamometer was used in the DDD.

To relate the reported joint forces to joint torques and healthy reference values, we translated the forces measured with a dynamometer to torques using an average forearm length of 26.5 cm and a combined upper arm and forearm length of 60.6 cm.^21,31^ On average, elbow flexion strength varied between 3 N and 18 N, which relates to estimated torque values between .8 and 4.8 Nm, about 2%–10% of the torques measured in a healthy reference population.^32^ Elbow extension strength varied between 3 N and 25 N (ie, .8–6.6 Nm), about 3%–22% of the torques measured in a reference population. Shoulder abduction strength varied between 3 N and 15 N (ie, 1.8–9.1 Nm), about 4%–18% of the torques measured in the healthy reference population.

The elbow flexion strength is barely sufficient for lifting the weight of the forearm and hand, which is about 2.5% body weight,^33^ approximately 15.9 N, and shoulder abduction strength is not sufficient to lift the entire arm, which is about 5.5% body weight,^33^ approximately 35.1 N with an estimated body weight of 65 kg.^34^

#### Range of motion

The pROM in shoulder abduction (elevation rotation in the frontal plane) is around 130°, which is about 30° less than in the reference population.^35^ Elbow flexion of DMD patients with Brooke Scale 4 is about 130°, compared to 150° in the reference population.^36^ Elbow extension is most limited in DMD patients. On average, DMD patients show a passive elbow extension of 30° of flexion, compared to the reference population who can, on average (hyper)extend the elbow about −5° of extension.^36^ Note that different methods for measuring ROM were used. Janssen et al.^3^ used 3D motion analysis to determine the aROM and pROM, while DDD used goniometry to determine pROM.

Regarding aROM, no movement is possible at the shoulder level, and for the elbow the aROM was similar to the pROM. The limited aROM can also be observed when looking at the reachable workspace. With a relative surface area (RSA) of .02 to .2, almost no shoulder movement is observed (a value of 1.0 corresponds to the envelope of the entire frontal hemisphere that the subject can reach).

#### Functional ability

Functional ability of the arms in DMD patients is usually measured with the PUL scale,^30^ see footnote^e^ Table 1. Table 1 shows that DMD patients in Brooke Scale 4 have no function left in the shoulder dimension. The scores in the elbow dimension are about 30% of the maximal possible score and only minor functional limitations are seen in the wrist and finger dimension.

#### Joint impedance

In DMD patients, the joint impedance, often referred to in the clinical field as muscle or joint stiffness, is elevated compared to the healthy reference population.^37–39^ At some point in time during the progression of the disease, the muscle strength becomes too low to overcome the elevated joint impedance in an extensive range of the functional workspace.^38,40^

The term joint impedance describes all the mechanisms in the joint that contribute to the resistance of motion,^41,42^ including all motion-dependent effects such as stiffness or non-elastic forces (ie, pose dependent), viscosity or damping (ie, velocity dependent), and inertia (ie, acceleration dependent).^42^ Joint impedance results from passive components (ie, biomechanical properties such as tendons, tissue, and inertia) and active components (ie, muscle reflexes or neural-driven contractions).^42^ The elevated joint impedance experienced in DMD presumably results from the passive components, such as shortened muscles, high levels of connective tissue, and joint contractures majorly developed by disuse and fibrosis.

Lobo-Prat et al.^38^ and Ragonesi et al.^37^ identified the combined passive joint moments (eg, weight and passive joint impedance components) in NMD patients for arm support applications. Lobo-Prat et al.^38^ showed a great improvement in the vertical and horizontal workspace with passive joint impedance compensation with respect to solo weight compensation in a DMD patient (Brooke scale not mentioned). Especially in combination with the low muscle strength in DMD, this passive joint impedance becomes an important factor to consider when developing the control of an arm support.

Three other studies^11,39,43^ report an increased (experienced) joint stiffness in DMD determined with varying methods. According to the results of Cornu et al.,^39^ the mean total joint stiffness is ∼20 times higher in DMD (Brooke scale not specified, age range 9–21 years) than that of healthy children. Measured during fast (4–12 Hz) sinusoidal perturbations (3°) to the right elbow during an active task (35%–75% maximally voluntary contraction). They state that the total joint stiffness increases exponentially with disease progression.^39^ Moreover, Lacourpaille et al.^43^ concluded that the index of muscle stiffness, measured by shear wave elastography using ultrasound, was significantly higher (up to 136%) in DMD patients (Brooke scale not specified, age range 8–23 years) compared to healthy controls). Moreover, Janssen et al.^11^ found that the experienced stiffness increases throughout the stages of DMD with a substantial increase in the late non-ambulatory stage, which includes DMD patients with Brooke Scale 4.

The results indicate that joint impedance increases over disease progression in DMD and that the passive joint impedance is a relevant component to consider in arm support compensation strategies.

#### Anthropometry

The intended target population’s body dimensions might deviate from the healthy reference population and should be considered when developing an arm support. DMD patients are prone to have a higher body mass index (BMI) and be overweight or obese.^44,45^ In addition, it is known that children with DMD exhibit a different growth pattern and are typically smaller than the healthy reference population.^46,47^ Deviating sizes are expected for the arm length, arm circumference and shoulder width. Besides the fitting, this might influence the position of the centre of mass (COM), affecting kinematic arm models.

#### Comorbidities and medication

Regarding the design of an arm support, important comorbidities should be considered. Scoliosis (an aberrant curvature of the spine) is common in DMD patients. Scoliosis leads to a skewed posture and a worse sitting balance; this might influence both the fitting and the effectiveness of an arm support. In addition, DMD patients have decreased bone mineral density, which commonly leads to fractures,^48^ so high loads exerted on the bones should be avoided. Finally, based on clinical observation, it is important to take the occurrence of shoulder subluxations into account, by limiting extreme shoulder ROM.

Another factor that should be considered is medication use (ie, corticosteroids). Many DMD patients use an intermittent corticosteroid regime (10 days on and then 10 days off). Although this has not been studied, patients anecdotally report more functional difficulties and muscle weakness during the 10-day off period. These variations should be taken into account when developing the control and level of support of an arm support.

### Functional requirements

#### High-level functional requirements

The DMD Upper Limb Patient-Reported Outcome Measure (DMD UL PROM), not to be confused with pROM (‘passive range of motion’), is an outcome measure used in DMD to track upper limb function decline in ADL. It includes activities that are identified as meaningful in the daily lives of people with DMD and impact their quality of life.^49^ We used the DMD UL PROM to define the high-level functional requirements. Authors SF and MJ ranked the DMD UL PROM activities on the required workspace and required strength needed to perform the activity, according to the scores presented in Table 2.

**Table 2.** Workspace and strength scores.

Score	Workspace (cm)	Strength (g)	Category
1	Small movements in horizontal plane (<10)	Hand movement with finger pressure <50	Must
2	Large movements in horizontal plane (>10)	Forearm movement no/weight <50	Must
3	Movement in sagittal plane close to body	Forearm movement with weight <100	Must
4	Movement in sagittal plane far from body	Entire arm no/weight <50 or forearm with weight <200	Should
5	Movements that require trunk movement	Entire arm with weight <100 or forearm with weight >200	Could
6	-	Entire arm with trunk no/weight <200	Could
7	-	Entire arm with trunk with weight >200	Could

**Figure 3.**
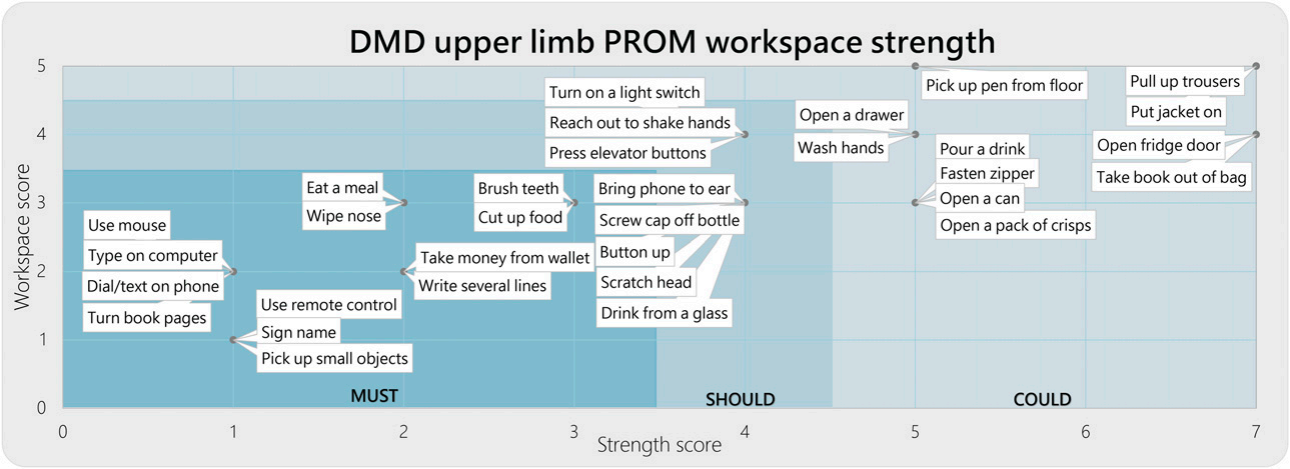
Classification of DMD UL PROM activities in the functional *must*, *should*, *could* requirement categories.

Next, we classified these activities into a ‘must,’ ‘could’ and ‘should’ category, see Figure 3. Where ‘must’ defines the requirements necessary for arm supports to assist the most feasible ADL. ‘Should’ describes the recommendable requirements that would increase the usability to gain function in the less feasible but important ADL, potentially to the drawback of increased complexity and bulkiness of the device. Where ‘could’ describe the nice-to-haves but hard to realise functional ADL gains.

According to our categorisation based on the workspace and strength scores, activities with movements in the horizontal plane, such as tabletop activities and movements close to the body in the sagittal plane while lifting small objects (<100 g), fall under the ‘must’ category. Reaching movements, such as pressing an elevator button or putting on a light switch, which requires lifting the entire arm in the sagittal plane far from the body or lifting the lower arm with medium weight (<200 g), fall under the ‘should’ category. Finally, arm movements that require high forces to lift heavy weight (>200 g), manipulate objects (ie, open a can, open a door, or drawer), or require trunk movements (ie, picking up pen from floor) fall under the ‘could’ category.

#### Range of motion

**Figure 4.**
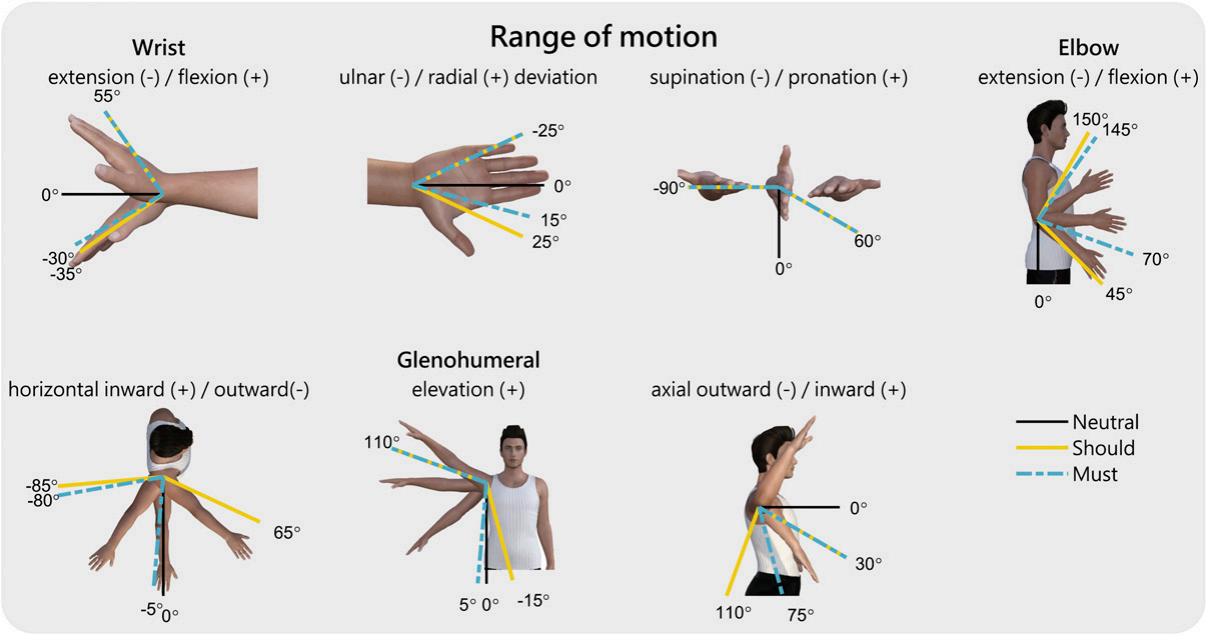
Indication of the range of motion of the wrist, elbow and glenohumeral joints required for the *must* and *should* categories. Note: The data in this figure is based on the ADL activities included in the DMD UL PROM items [77, 78], including: (*must*) turn book pages, eat a meal, wipe nose, brush teeth; (*should*), drink from a glass, bring phone to ear, scratch head, button up, press elevator buttons, turn on a light switch, reach out to shake hands. The values are rounded to 5°.

For the ROM analysis, we looked into studies that analysed the required ROM for daily activities. We searched for the activities of the DMD UL PROM items and presented the largest ROM required for the ‘must’ and ‘should’ categories, see Figure 4. Since the activities in the ‘could’ category were hardly reported in the literature, the found values gave an underrepresentation of the actual ROM needed in the ‘could’ category. Moreover, the required trunk movements were not reported, and therefore, the ‘could’ category was excluded from this analysis.

#### Velocity

Limited literature was found on movement velocities for the DMD UL PROM activities. Two studies were identified that reported the angular joint velocity during ADL in the healthy reference population. According to Rosen et al.,^50^ the upper extremity movement velocities measured in four activities (ie, arm reach to head level, move object at waist level, pick up phone on wall/hang up, and eat with spoon) ranged between −141 and 172°/s for the shoulder joint, and −172 and 145°/s for the elbow joint. The mean velocity over the four activities was ±85°/s for the shoulder, and ±93°/s for the elbow joint. Karner et al.^51^ report the average and peak angular velocity over four ADL (eg, combing hair, drinking from bottle with straw, interacting with own body, and move other hand). They found 101°/s (peak 228°/s) for the elevation rotation, 34°/s (peak 82°/s) for horizontal rotation, 83°/s (peak 134°/s) for axial rotation and 98°/s (peak 181°/s) for elbow flexion.

More research is required on the movement velocity to determine the functional requirements in a ‘must,’ ‘should’ and ‘could’ categorisation. We think it is more important to give back independent task execution at an albeit lower but stable and predictable movement velocity than moving fast on a natural arm velocity equal to the healthy reference population. Moreover, too quickly might feel unsafe, but too slowly may lead to frustration.

#### Support level

The required support levels need to be known to be able to choose the appropriate actuation (eg, type, size and power). The results of the simplified analysis to estimate the static kinematic joint moments of the human shoulder and elbow joint required in movements of the ‘must,’ ‘should’ and ‘could’ categories are displayed in Table 3. We looked at a tabletop and feeding activity for the’ must' category. When taking the maximum values, an internal joint torque of approximately 2 Nm is generated in the elbow and 5 Nm in the shoulder joint (elevation rotation). For the ‘should’ category, we looked into a ‘reaching at top of head level’, ‘bring an object (<200 g) to head level’ and ‘reaching at shoulder level’ pose. Approximately −2 Nm (extension moment) is generated with the elbow and 11 Nm with the shoulder joint (elevation rotation). For the ‘could’ category, approximately 3 Nm with the elbow and 13 Nm with the shoulder joint is required to ‘reach at shoulder level while holding an object (200 g)’. This activity is meant to correspond to ‘wash hands,’ ‘open drawer’ or ‘open fridge door.’ The other DMD UL PROM activities in the could category, such as ‘pick pen from floor,’ ‘take book out of bag,’ and ‘put jacket on,’ require a substantial trunk inclination angle, which was not considered for this analysis.

**Table 3.** Net shoulder and elbow joint moments in Nm for six ADL activities.

Joint moment [Nm]	Must		Should			Could
Activity	Tabletop activities 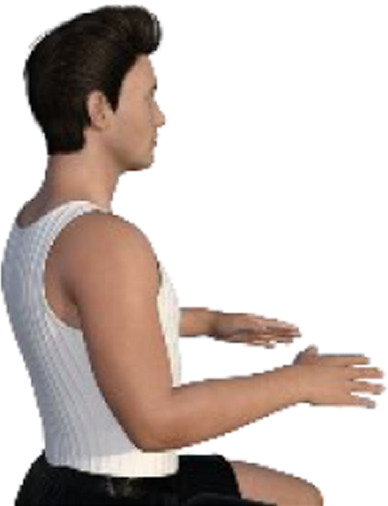	Feeding activities(50 g) 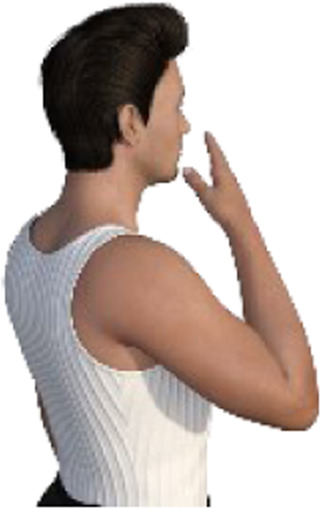	Reach top head 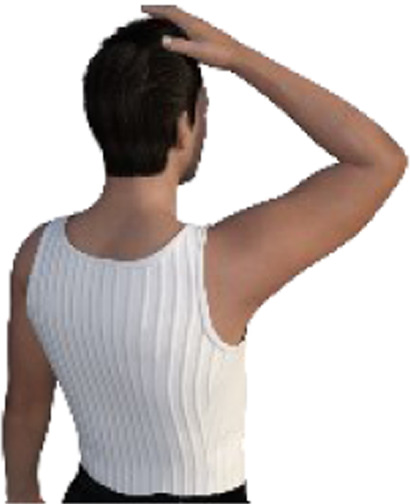	Bring object (200 g) to head 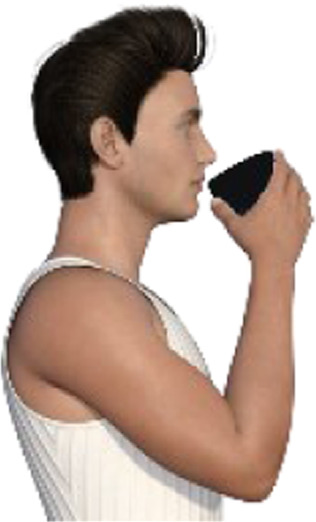	Reach shoulder level 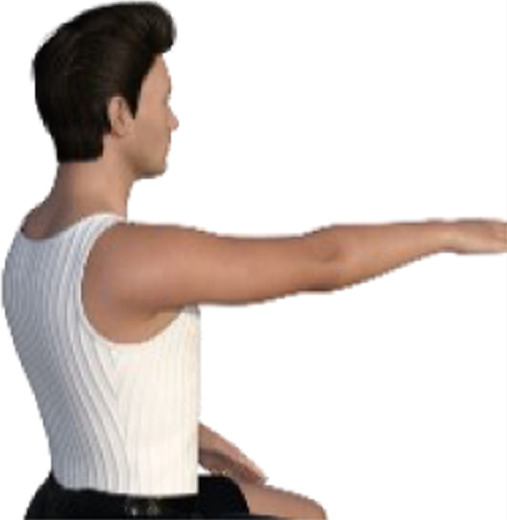	Reach at shoulder height with object (200 g) 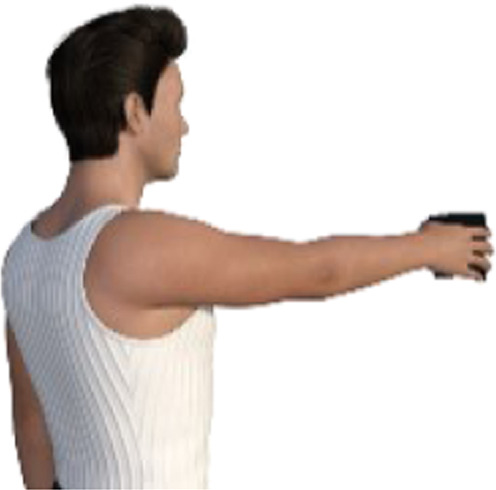
Elbow shoulder	2.3	1.1	−1.8	−0.3	-	3.1
Horizontal	-	-	-	-	-	-
Elevation	3.2	5.4	6.2	7.4	11.2	12.5
Axial	0.8	1.3	1.2	2.2	-	-

Model representing activities retrieved from DAZ Productions.^52^

These joint moments are in accordance with the findings of Karner et al.^51^ However, it should be noted that the numbers presented here are only rough estimations to indicate the required support level. These calculations are based on static poses of a dynamic movement, so the moments of inertia are not taken into account. However, considering the relatively low speeds and angular accelerations, the expected relative contribution is fairly limited. Additionally, the weight of the device is not considered since this depends on the technology but should be considered when choosing the appropriate (actuation) technology. Furthermore, the additional joint moments required to overcome the (elevated) passive joint impedance must be considered. Lobo-Prat et al.^38^ and Ragonesi et al.^37^ concluded that passive joint impedance is a relevant component and that the arm dynamics cannot be modelled by a simplified gravitational kinematic model alone. For proper compensation of the passive joint impedance, it is essential to know its behaviour over the pROM among DMD Brooke Scale 4, either generalised or personalised. Unfortunately, these studies^37,38^ do not provide enough quantitative data on the level and behaviour of the passive joint impedance and are therefore not included to define the required support level.

### Technical requirements

The technical requirements are divided into four categories: *mechanical structure*, *actuator technology*, *control approach*, and *human interface.* The requirements are expressed in Table 4. Each category is subdivided into ‘*performance*’ and ‘*safety*.’ Supplementary details to the table are given below.

**Table 4.** Technical requirements.

Requirements	Should/Could	Must	Source
**Mechanical structure**			
*Performance*			
Anatomy	Follow the human anatomical structure^1^	Allow 3D motion in shoulder and elbow joint	^1^ Dunning et al.^63^; Essers et al.^64^
DOF	Could: Allow trunk movements^1^ 5 DOF (eg, glenohumeral horizontal, elevation, axial rotation, elbow extension/flexion and forearm supi-/pronation) Include (passive) dynamic wrist support	4 DOF (no glenohumeral axial rotation or forearm supi-/pronation) Include static passive wrist at rest position	^1^ Cruz et al.^29^ Dunning et al.^63^; Essers et al.^64^; Gull et al.^65^
Singularity	Prevent singularity by design eg, 1) configure singularity outside the movement range, 2) use redundant linkages, or 3) optimise the length of the linkages	Solve for singularity (eg, by end-stops, control limits)	Castro^66^; Perry et al.^67^
Joint alignment	Misalignment <3.5 cm^1^ Implementation of a form of passive or active misalignment adaptation (ie, self-aligning) joints^1,2^	Misalignment <10 cm Note: 10 cm joint misalignment can result in high interaction torques (<±1.46 Nm) or forces (<±230 N)^3^	^1^ Otten et al.^53^ ^2^ Wu et al.^68^ ^3^ Schiele and van der Helm^55^
Fitting	Adjustable fitting for 95% of intended target population, eg, fully adjustable to (upper) arm length, circumference, shoulder width, hunchback angle	Use of multiple sized components, eg, small, medium, large	
Obtrusiveness	Not wider than an electric wheelchair (±700 × 1500 mm) No voluminous components at radial or ulnar side of forearm and frontal side of the upper arm	Passes through doorway No hinder in arm relaxation, eg, clash with tabletop	Essers et al.^64^
Outdoor use	Device protected from rain and dust (IP66) and robust to oscillations and collisions to environment	Usable inside and robust to oscillations and collisions to environment	Landsberger et al.^69^
*Safety*			
Angle limits	Personalised hardware end-stops to limit device ROM to user pROM	Device should not exceed pROM of user	
Sharp edges, skin pinching, hair entanglement	No scissor mechanisms or pinch points by design, and protection of rotating parts	No sharp edges, mitigation measures to prevent risk of pinched skin or hair entanglement	
**Actuator technology**			
*Performance*			
Maximum motor torque	20 Nm	>10 Nm^1^ Note: Up to 12.5 Nm required for human arm in ADL^2^, additional torques are required to move the device itself.	^1^ Otten et al.^53^ ^2^ Table 3
Motor torque bandwidth	100 Hz	40 Hz	Otten et al.^53^
Delivered torque resolution	<.5 Nm	<1 Nm	Stienen et al.^60^
Movement intention detection directions			
Force-based	Joint torques accurately measured for movement intention in all assisted joints (impedancecompensation-based)	A 6DOF force sensor at each interface point to determine *x*, *y*, *z* intention direction (admittance control)	
sEMG-based	Joint angles/torques estimated from muscles that contribute to the supported motion using pattern-recognition or regression-based algorithms	Three agonist/antagonist pairs muscles for *x*, *y*, *z* direction	Lobo-Prat, Kooren, and Janssen^61^
Movement intention detection accuracy			
Force-based (interface-level)	Force sensors used for admittance control with DMD are: - ATI Industrial Automation Mini45 Force/Torque sensor, measurement resolution of ¼ N^1,2^ - Nano 25, ATI Industrial Automation, Apex, USA^3^	No clear measured resolution requirements were found for interface force-based methods (eg, admittance control), therefore the used force sensors used in literature are reported in the could/should column.	^1^ Corrigan and Foulds^70^ ^2^ Lobo-Prat et al.^38^ ^3^ Kooren et al.^71^
Force-based (joint-level)	0.1 Nm (measured torque resolution on joint level)	<1 Nm	Stienen et al.^60^
sEMG-based	SNR <25%	SNR <50%	Lobo-Prat et al.^61^
*Safety*			
Movement velocity [°/s]	Glenohumeral: −66 to 59 Elevation with 0° horizontal rotation −68 to 76 Elevation with 90° horizontal rotation −95 to 90 Axial rotation Elbow: −89 to 83 Extension/flexion	Give independent task execution back while moving at a safe and predictable speed.	Data present mean ADL angular velocities based on the recorded kinematic data reported in Rosen et al.^50^
Temperature	< skin temperature	<48°C^1^	^1^ IEC 60601-1:2005 standard
Audible acoustic energy	<30 dBA^1^	<80 dB^2^	^1^ Kooren, Lobo-Prat and Keemink^71^ 2 IEC 60601-1:2005 standard
**Control approach**			
*Performance*			
Support gain	Gain optimised over ROM, tuneable support gain depending on variable needs on daily basis (eg, fatigue, morning stiffness, temperature)	Gain similar over ROM, no static floating in space when relaxed (ie, 80% > gain <100%)	Essers et al.^64^; Kooren et al.^71^
Compensation strategy	Detect and compensate for additional load of grasped object in hand. Distinguish between interaction with environment (eg, table, caregiver push against arm) and users’ movement intention	Distinguish between voluntary movement and intrinsic passive joint impedance^1^	^1^ Lobo-Prat et al.^38^
Transparency	Residual actuator impedance <.3 Nm	Residual actuator impedance <.4 Nm (50% of elbow flexion strength, ca .8–4.8 Nm)^1^	^1^ Muscle strength section
*Safety*			
ROM limits	System ROM = pROM	System ROM < pROM user Prevent hardware-to-hardware collisions Prevent singular configurations	
Torque limits	More research required to find safe general torque limit within the pROM	Subject-specific limit determined at device provision; physiotherapist checks stretch values for pROM	
Velocity limits	Predictable and stable at ADL movement velocities.	Predictable stable velocity < ADL velocities	More research required
**Human interface**			
*Performance*			
Donning/Doffing	<10 min a day	<15 min	Kooren et al.^71^
Comfortability			
Usage duration	>8 h/day	>4 h/day	
Skin pressure	Pressure on skin:^1^ Upper arm: 21.6 ± 8.7 mmHg Forearm: 20.1 ± 7.7 mmHg	Pressure on skin:^2^ < 30 mmHg No stiff material on top of a bony structure to prevent pressure points	^1^ Schiele and van der Helm^55^ ^2^ Pons^72^
Shear forces	No shear forces	No sliding in interface on skin	
*Safety*			
Risk of fall-out	No sliding in the interface	The arm of the user should not be able to fall out the interface in all possible configurations	
Fire-proof	Fabrics and material used are fire resistant	Fabrics and materials used do not melt or catch fire when in contact with fire	16CFR1610: standard for the flammability of clothing textiles
Biocompatible and hygiene	Easily detachable and robust to was-/dry-machine	Skin-friendly, breathable, and detachable to wash	ISO 10993-1:2009
Quick release	Intuitive interface quick release design that any bystander could use in case of emergencies	User interface attachments should be quickly releasable <30 s by trained caregiver that knows how to handle the arm support that stays nearby the user to assist in case of emergencies	

3D: three dimensional; DOF: degrees of freedom; IP: international protection; (p)ROM: (passive) range of motion; sEMG: surface electromyography; DMD: Duchenne muscular dystrophy; SNR: signal to noise ratio; ADL: activities of daily living.

#### Mechanical structure

The mechanical structure should not add additional load to the spine to prevent deterioration of potential scoliosis, a frequent comorbidity in DMD, since trunk muscles are also weakened. Therefore, it is recommended to connect the arm support to the (electrical) wheelchair to carry the weight of the device.

Moreover, the mechanical design of the device must not restrict the user’s already limited pROM^53^ and allow the ROM required for the in the ‘must’ and preferably ‘should’ defined categories. However, it is also crucial to be aware of the aforementioned joint contractures, which people with DMD suffer from. To assist the ADL in the ‘could’ category, the mechanical structure should also allow for trunk movements. This is important for the large workspace reaching tasks.^29^

Moreover, the arm support should not be obstructive, eg, it must fit through a standard-sized doorway.^29^ Preferably it looks slender and slim instead of bulky and stigmatising.^7^

Finally, the mechanical axes must be optimally aligned to the human joints because joint misalignment can result in high interaction forces and injury.^54–56^ There will always be some degree of joint misalignment since human joints are not pure revolute joints, have complex geometries, and the axes of rotation translate during rotation.^56^ To prevent adverse events, misalignment can be limited by design, eg, self-alignment mechanisms or compliant actuators.^53,54^

#### Human interface

The human interface that transmits the forces from the exoskeleton to the body must be comfortable and fit the dimensions of the individual user. A well-balanced consideration should be made between comfortability (eg, skin pressure, shear forces, displacements) and safety (eg, sliding or falling out, skin irritation, bruises, or discomfort). Preferably, the interface is easily personalised, detachable and easy to clean (eg, washing machine-resistant and cleanable surfaces).

For the user’s independence and compliance, the device should be easy to donn and doff with the help of a caregiver, but preferably independently by the user itself. Note that the aforementioned joint misalignments between the human and system joints could also result in displacement and shear forces at the human interface.^55,57^ This should be minimised and checked during donning.

#### Actuator technology

Based on required joint torques and the limitations mentioned above of the (semi-)passive systems, a form of motorised support for the intended target population will be needed. For a motorised arm support, a choice should be made considering the type and placement of the actuator technology. The actuator type can vary from electrical motors (eg, servo, step motor, series elastic actuator) to hydraulics or pneumatic (artificial muscles).^58,59^

For the placement of the actuator technology, a choice between directly on the joint or teleported (ie, externally positioned), such as cable-driven systems, can be made. The advantage of directly placing the actuator on the joint is that the design can be simple since no transmission mechanisms are required. The disadvantage is that a heavy and more distally placed motor negatively influences the mass distribution, ie, effective weight and inertia.^58^ Moreover, multiple actuators around a single joint negatively affect the device ROM (eg, colliding motors at the shoulder joint for example). The advantage of teleported actuators, such as cable-driven systems, is lower limb inertia and enlarged ROM. However, the disadvantage of cable-driven systems is that for a bi-directional motion, two cables and two motors are required (eg, cables can only pull) and that the cables introduce (non-linear) friction.^58,60^

#### Control approach

Preferably, the device supports the arm naturally and intuitively without the use of the contralateral arm or pre-defined trajectories but must detect the movement intention from a (physiological) signal that is intuitively related to the supported motion.

Control interfaces such as surface electromyography (sEMG) and force-based interfaces are promising strategies for achieving fine control movements.^40^ With sEMG control, the muscle activity of selected muscles indicates the user’s movement intention. Often, the agonist and antagonist muscles are used for opposite movements, for example, the m. biceps brachii and m. triceps brachii for elbow flexion and extension. The options with the force-based approaches are broad, from admittance control^26,38^ to impedance compensation-based (eg, of weight and passive joint impedance) approaches. With admittance control, a force sensor is used to measure the interaction forces between the user and device at interface level to measure the movement direction and intention of the user. The forces are then translated into a movement of the arm. With impedance compensation-based methods, the required support torques are determined on a joint level, where the orientation of the arm determines the level of support.

Lobo-Prat et al.^40,61^ compared sEMG and force-based admittance control interfaces in adults with DMD. They concluded that sEMG-based control was perceived as less fatiguing but force-based control as more intuitive since force-based control is closer to the natural way of interacting with the environment. They recommended the use of force-based control interfaces for people with more voluntary forces and sEMG-based for people where voluntary forces are below the intrinsic forces (eg, weight and passive joint impedance) of the arm. This aligns with their findings that the participant with Brooke Scale 4 preferred the force-based methods, while participants with Brooke Scale 5 and 6 preferred the sEMG interface. With sEMG-based interfaces, it is easier to distinguish between voluntary movement intention and the intrinsic forces of the arm.^61^ However, sEMG-based interfaces have the practical drawback that it is a difficult and time-consuming installation, due to the sensitivity of proper electrode placement. Moreover, it can become uncomfortable to have multiple electrodes in contact with the skin for a longer period, and long-term sEMG measurement stability is poor.^26,61^ Although both methods have pros and cons, based on Lobo-Prat et al.,^40,61^ we recommend force-based methods for people with DMD Brooke Scale 4 since this is reported as more intuitive and has practical advantages.

#### Safety

Obviously, the device must be safe for the user and bystanders. The mechanical design should be strong and stiff enough to prevent bending (which can result in control issues) and breakage, also considering unintended usage. Considering the actuator placement, the configuration should not allow for singularity (ie, configurations where the actuators mechanically get ‘stuck’).

Audible acoustic energy should be considered when choosing the actuator technology. Most types of actuators make noise, while the shoulder actuators might be placed close to the ear. The system should not hinder communication with others, let alone the risk of hearing damage [IEC 60601-1:2005 standard]. Similarly, the electric magnetic radiation of the actuator technology should be considered. This might affect active implants (eg, pacemakers) of the user or bystander [IEC 60601-1-2:2014 standard].

The device should move as the user intended, which is an important safety aspect for selecting the control approach. If the robot moves differently, it can cause an unsafe situation, eg, spilling hot water over its own or bystander’s skin. It is expected to have higher precision with impedance compensation-based approaches over sEMG approaches.^61^ Other vital safety aspects for the control software are to warrant the torque, velocity and angle limits. Overstretching due to high torque, unexpected fast movement, or movement outside of the pROM of the patient could lead to trauma of soft tissue, such as (shortened) muscles and ligaments, or even damage to the cartilage and bones. Stretching the joints with elevated joint impedance can be beneficial,^62^ but care should be taken with stretching beyond the rigid contractures. From consultation with clinicians, stretching exercises performed by therapists can break a bone in patients with severely reduced bone mineral density. Unfortunately, the patient is not always aware of the reduced bone mineral density. Therefore, it is crucial that the ROM of the device does not exceed the pROM of the user. Since the level of contractures, and thus the pROM, varies among the target population, user-specific end-stops are recommended to limit the device ROM. Physical hardware end-stops should be provided to prevent overstretching of the human joint in case of a software error or unexpected behaviour. Moreover, it is recommended to identify the maximal allowable joint torque at the joint limits that are comfortable to the user before using the device.

Furthermore, end-application restrictions should apply, eg, the device should not be used in people with involuntary movement intentions such as spasticity or epilepsy. Additional safety requirements are expressed in Table 4.

## Discussion

This paper provides the functional and technical design requirements of wearable assistive arm support technology for people with DMD Brooke Scale 4.

The clinical characteristics show that the intended target population of DMD presents severe muscle weakness, with muscle strength of approximately 2%–22% of the healthy reference population. On average, their functional ability without arm support is limited to tabletop activities because of their severely impaired shoulder function. However, some variation in functional abilities is present, along with a great variety in the level of joint contractures, arm circumferences, and BMI among patients. This implies that a certain level of individual customisation is necessary.

The functional requirements show that activities with light weights (<100 g) close to the body, such as computer(gaming), personal hygiene, feeding activities (incl. drinking with a straw), and writing, are a ‘must.’ Activities further away from the body or with heavier weights (<200 g), such as turning on a light switch, scratching your head, or drinking from a glass, fall under the ‘should’ category. Activities requiring trunk movement for reaching and lifting heavy weights (>200 g) fall under the ‘could’ category and are less feasible to realise. Considering the support level requirements, a form of motorised support is preferable for the intended target population. The advantage of a motorised system is that it can automatically adjust to the required compensation levels at different heights in the workspace (which is not yet the case in the semi-active systems), it allows for passive joint impedance compensation, and theoretically is able to detect and compensate for additional lifted objects. Moreover, it will enable the user to tune the level of support to a level that is required and feel comfortable, this level might vary between or even during the day(s) depending on the fatigue levels. It may need some experience for the user to find the right balance between sufficient training of the arms and preventing the risk of overuse.

Unfortunately, there are not enough quantitative data available on the level and behaviour of the passive joint impedance over the pROM in DMD Brooke Scale 4 yet. Therefore, further investigation of the behaviour (eg, position, velocity, acceleration dependence) of the (elevated) passive joint impedance in DMD and whether it can be captured in a generalised model or should be personalised is needed. Furthermore, no clear literature was found on acceptable torque and velocity limits that are safe and comfortable for the user. Follow-up studies should examine safe levels for the torque and velocity limits. It is expected that the performance of the control of the device (eg, robustness, predictability, and safety) affects the acceptable movement velocity that feels safe to the user. Finally, no standards are yet available to quantify the comfortability of existing arm supports,^57^ making it difficult to compare and construct design requirements. However, within this paper, we have provided an educated guess for these requirements to highlight their importance because safety and comfort remain critical aspects of user acceptance. Future studies could aim to compare existing arm supports on comfortability.

The limitations are a lack of available data and literature concerning the target population and the requirements. In the case of limited literature, we verbally and by email consulted with three clinicians specialised in DMD (physiotherapist, occupational therapist and paediatric rehabilitation physician) to get more clarification on some of the clinical topics. Moreover, several assumptions had to be made to interpret reported values to be relevant for this paper.

Although the narrative focus is on DMD Brooke Scale 4, the concluding design requirements might also apply to other pathologies with a similar functional profile (eg, other muscular dystrophies, stroke, spinal muscular atrophy, or amyotrophic lateral sclerosis) or more severely affected DMD (eg, Brooke Scale 5). However, the importance of starting with specific patient needs should be stressed to ensure a good match between needs and design. The target population can be expanded after the functional gain and compliance is proven. In this way, the device is fit to the user instead of fitting the user to the technology, a commonly seen pitfall in assistive technology companies.^17,73^

One of the biggest challenges in the design of a (motorised) arm support is to improve the arm function without limiting the residual function of the user. Since the technology needs to encompass the arm, the design space is limited, while high support torques are required, making it hard to render the design into a slender construction. Moreover, arm supports (eg, orthotics) deal with the residual arm function of the user that can vary among users. It is essential not to restrict the residual function to prevent further function loss or abandonment of the device. Moreover, the extreme muscle weakness of the DMD population increases the complexity of the control approach and the required safety measures. Another challenge is finding the right balance between adding complexity to the assistive technology and the user’s gain in functionality. To prevent technology-driven overdesign and reduce the technology’s complexity and costs, we categorised the requirements in a ‘must,’ ‘should,’ and ‘could’ category and recommend starting with a minimal viable product that supports the ‘must’ requirements. When sufficient functionality and acceptance are proven, the system is ready to implement more complexity for the next iteration. Although it is challenging to fulfil the needs of the target population, they are expected to benefit significantly from motorised arm supports to assist arm function. As the disease progresses, arm supports are expected to slow down the functional loss by the involvement of the arms as daily practice, which is beneficial for muscle maintenance, bone mineral density,^74^ and prevention of contractures caused by disuse.^20^ Nevertheless, arm supports can provide more independence, social participation and, thereby, improve their quality of life.

In addition and beyond our scheme, other personal and environmental barriers (eg, awareness, acceptance, financial situation, the device provision process, a lack of follow-up procedures, and coordination between service and funding) should be tackled. These factors will differ between counties. Unfortunately, motorised arm supports’ expected (development) costs are high. On the other hand, using arm supports can also reduce healthcare costs due to the aforementioned clinical benefits. A large study on the effectiveness and cost-effectiveness of assistive technology for impaired arm function is currently ongoing in the Netherlands, and results are expected in the upcoming years.^75^

This work will be followed up by developing a dedicated assistive arm support based on the identified requirements. Although multiple solutions are possible from the specified requirements, we expect a motorised arm support with intuitive force-based weight and passive joint impedance compensation best match the needs of the DMD Brooke Scale 4.

## Conclusions

In the development process of assistive technology, it is essential to start with the specific needs of the intended user. People with DMD Brooke Scale 4 have severe muscle weakness (<22% arm strength compared to the healthy reference population), which leads to severe functional impairments, with almost no active movement in the shoulders and limited movement in the elbow. This paper categorises the functional requirements for assistance arm supports in people with DMD Brooke Scale 4 into a ‘must, ‘should’, and ‘could’ category and links this to the technical requirements. A form of motorised actuator technology with intuitive movement intention detection is recommended because it allows the implementation of control algorithms to adjust for the correct workspace height, allows for passive joint impedance compensation, can adapt to muscle fatigue and can compensate for the additional weight of lifted objects. Due to the severe muscle weakness, this population is vulnerable, and extra care should be taken with the safety considerations raised in the technical requirements. The design must not limit or restrict the residual function of the user nor increase the risk of injury. This paper can be used for the development of arm supports for people with DMD Brooke Scale 4 and make them more user-centred.
